# Supplementary figures and images for: A peptide derived from sorting nexin 1 inhibits HPV16 entry, retrograde trafficking, and L2 membrane spanning
Source: Tumour Virus Res. 2024 Jun 21;18:200287. doi: 10.1016/j.tvr.2024.200287 (PMC11255958; doi:10.1016/j.tvr.2024.200287)

**Supplemental Figure 1**


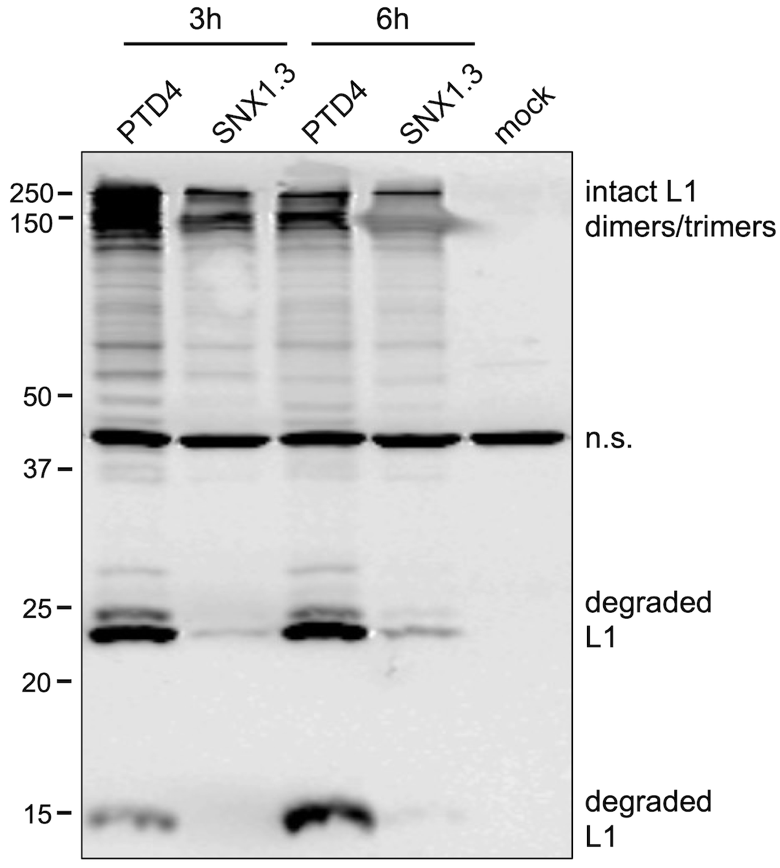


**Supplemental Figure 2**


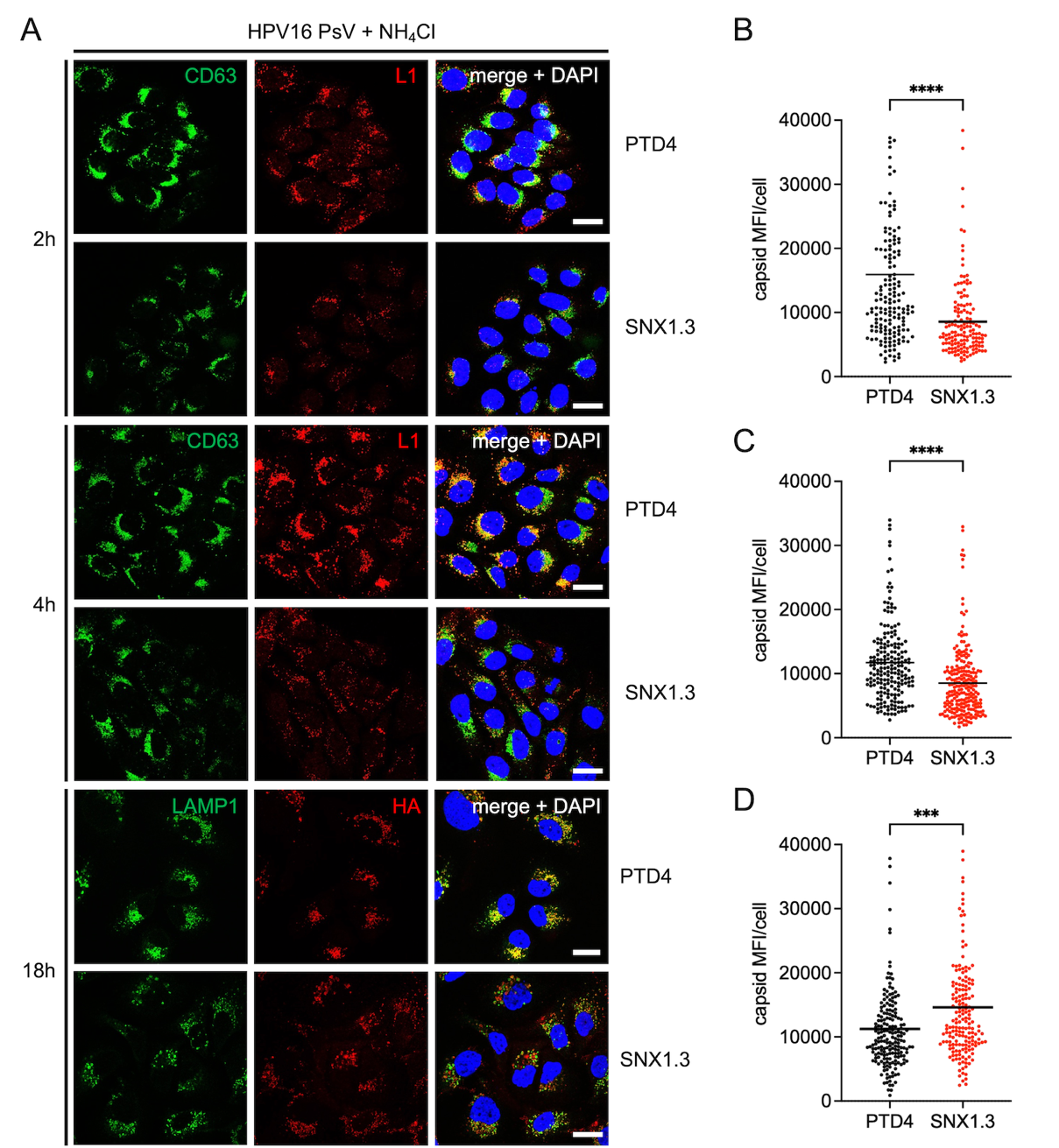


**Supplemental Figure 3**


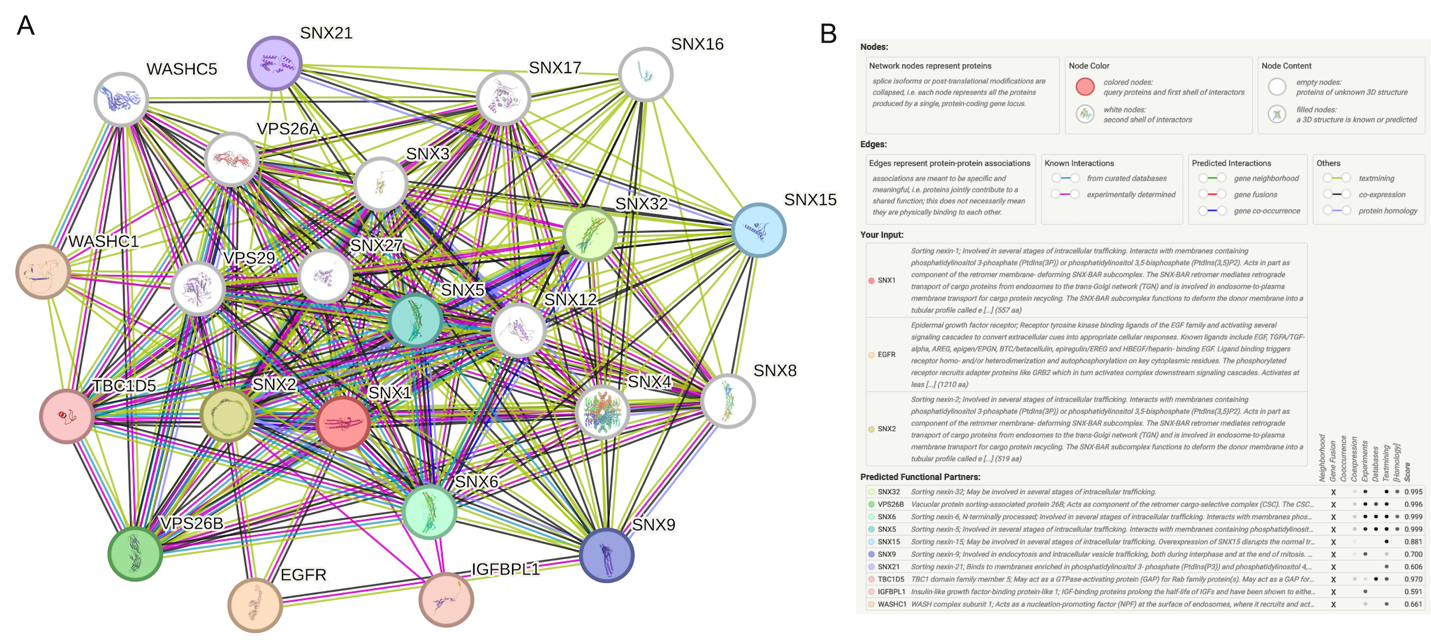

Supplement: Multimedia component 1 [file mmc1.docx]
